# Discovery of a New Cave in Kentucky

**Published:** 1849-10

**Authors:** 


### DISCOVERY OF A NEW CAVE IN KENTUCKY.

Professor Horsford read to the Scientific Association, at the late meeting in Cambridge, the following account of a new cave found in Kentucky:

This cave was discovered, a few weeks ago, in Kentucky, about twelve miles distant from the celebrated Mammoth Cave, and a few miles from Bowling Green, at the mouth of Green river. The discoverer, Mr. S. G. Stevenson, of the latter place, informed me that he had penetrated five miles, where the cave was still extending; and it is more than probable that it will prove the largest cave known. It abounds in a variety of beautiful avenues, and the stalactites and stalagmites, and other incrustations, are there found in every form. A few small samples were inclosed in a letter to me; the one proves by analysis, to be a sulphate of alumina, and the other a colcothan. The alumina does not contain either the potassa nor the ammonia, but forms a double salt with the soda; although I have not definitely ascertained the existence of the base (soda). I have satisfied myself that it does not contain either of the other bases, and that, from its permanent crystalline condition, it cannot be a pure sulphate of alumina, without a double base—this forming a deliquescent salt; also, from the fact that the whole country abounds in saline springs, brings the conclusion that it is a soda-alum. Within two miles from the new cave a regular stratum of this soda-alum has likewise been discovered. I am in daily expectation of a collection of specimens, which I requested to be forwarded by express, not alone of all the minerals, fossils, and incrustations, but also of any reptiles or fishes that might be discovered in the cave; and regret much that they have not yet come, in season to present to the present meeting.

